# To look or not to look? Reward, selection history, and oculomotor guidance

**DOI:** 10.1152/jn.00275.2018

**Published:** 2018-07-18

**Authors:** Daniel Preciado, Jan Theeuwes

**Affiliations:** Department of Experimental and Applied Psychology, Vrije Universiteit Amsterdam, Amsterdam, The Netherlands

**Keywords:** antisaccade, attentional bias, eye-tracking, reward-driven, goal-driven

## Abstract

The current eye-tracking study examined the influence of reward on oculomotor performance, and the extent to which learned stimulus-reward associations interacted with voluntary oculomotor control with a modified paradigm based on the classical antisaccade task. Participants were shown two equally salient stimuli simultaneously: a gray and a colored circle, and they were instructed to make a fast saccade to one of them. During the first phase of the experiment, participants made a fast saccade toward the colored stimulus, and their performance determined a (cash) bonus. During the second, participants made a saccade toward the gray stimulus, with no rewards available. On each trial, one of three colors was presented, each associated with high, low or no reward during the first phase. Results from the first phase showed improved accuracy and shorter saccade latencies on high-reward trials, while those from the second replicated well-known effects typical of the antisaccade task, namely, decreased accuracy and increased latency during *phase II*, even despite the absence of abrupt asymmetric onsets. Crucially, performance differences between phases revealed longer latencies and less accurate saccades during the second phase for high-reward trials, compared with the low- and no-reward trials. Further analyses indicated that oculomotor capture by reward signals is mainly found for saccades with short latencies, while this automatic capture can be overridden through voluntary control with longer ones. These results highlight the natural flexibility and adaptability of the attentional system, and the role of reward in modulating this plasticity.

**NEW & NOTEWORTHY** Typically, in the antisaccade task, participants need to suppress an automatic orienting reflex toward a suddenly appearing peripheral stimulus. Here, we introduce an alternative antisaccade task without such abrupt onsets. We replicate well-known antisaccade effects (more errors and longer latencies), demonstrating the role of reward in developing selective oculomotor biases. Results highlight how reward and selection history facilitate developing automatic biases from goal-driven behavior, and they suggest that this process responds to individual differences in impulsivity.

## INTRODUCTION

One of the most often used tasks to investigate cognitive control is the antisaccade paradigm. In this task, an observer is instructed to make a rapid eye movement either toward (prosaccade) or away (antisaccade) from a peripheral visual stimulus. Typically, stimuli with an abrupt onset are used in this task, as they are known to automatically attract and capture attention and eye movements (Hallett 1978; Munoz and Everling 2004). Studies using this paradigm have established that antisaccades away from a stimulus with an abrupt onset have longer latencies and are more error prone than prosaccades toward the stimulus. Importantly, errors in this task are considered to result from failure to inhibit the automatic saccade, while longer latencies are considered to reflect the additional processing steps required to first inhibit the automatic, stimulus-driven prosaccade, and then to execute the voluntary antisaccade to an arbitrary location in the visual field away from the stimulus (Hutton 2008; Hutton and Ettinger 2006; Munoz and Everling 2004).

The ability to suppress a reflexive, stimulus-driven response to an abrupt onset and perform a controlled, volitional movement away from it reflects an important component of daily human activity, one that is regarded to be a crucial executive control mechanism underlying processes such as gratification delay, response selection, motor inhibition, and impulse control (Aron 2007; Eagle et al. 2008). Deficiencies in this ability have been implicated in a variety of mental and neurological disorders. For instance, while it is well known that healthy participants have error rates ranging from 5% to 25% (Reuter and Kathmann 2004), significantly longer latencies and higher error rates are reported for participants suffering from schizophrenia (Fukushima et al. 1988; Haraldsson et al. 2008; Larrison-Faucher et al. 2004; Levy et al. 2008), attention deficit and hyperactivity disorder (Hakvoort Schwerdtfeger et al. 2013; Klein et al. 2003; Oosterlaan et al. 1998), mood and anxiety disorders (Jazbec et al. 2005; Mueller et al. 2010, 2012), and in neurological patients with lesions in the frontal lobes (Guitton et al. 1985). From this perspective, it can be inferred that patients with disorders that affect frontal lobe function might have difficulties suppressing automatic prosaccades, which is taken to reflect a broader deficit in voluntary inhibitory control of behavior (Munoz and Everling 2004).

The interplay between the control processes necessary to execute a correct antisaccade, while suppressing the automatic, reflexive tendency to look toward a salient peripheral stimulus with an abrupt onset constitutes the basis of the antisaccade task. To investigate this interplay, most studies of attentional control mechanisms distinguish between endogenous, voluntary attentional selection driven by the goals and intentions of the observer, and automatic, exogenous control driven by the physical properties of a stimulus (Corbetta and Shulman 2002; Theeuwes 2010). By this distinction, prosaccades toward a stimulus with an abrupt onset are considered to result from an automatic, stimulus-driven orienting mechanism akin to a physiological reflex that is independent from the intentions, goals, and expectations of the observer (Neumann 1984; Posner 1980). In contrast, antisaccades reflect the activity of a deliberate, voluntary control mechanism that acts in tune with the explicit intentions, goals, and motivations of the observer (Carrasco 2011; Jonides 1981; Theeuwes 2010, 2018). Accordingly, performance differences between prosaccades and antisaccades in the classical antisaccade task can be considered to reflect the conflict between exogenous (automatic) and endogenous (voluntary) attentional and oculomotor biases.

However, the exogenous/endogenous dichotomy view has proven to be insufficient to account for a variety of attentional selection effects that cannot be explained solely in terms of stimulus- or goal-driven attention alone. Specifically, a third attentional control mechanism has been proposed, “selection history,” describing how the history of attentional selection and deployment can elicit lingering selection biases that are unrelated to the goals of the observer or the physical features and relative salience of a perceived stimulus. In this sense, selection history describes, for instance, how reiterated encounters with a particular stimulus lead to the recognition that its occurrence or location can be reliably predicted, or how a stimulus can become a potential signal of threat or reward for the observer (Awh et al. 2012; Failing and Theeuwes 2017; Le Pelley et al. 2016). Indeed, different studies have demonstrated that merely by presenting a stimulus more often at one particular location is enough to modulate attentional selection. For instance, it has been demonstrated that whenever a singleton distractor was presented much more frequently at one particular location than at any other, attentional capture effects elicited by a distractor presented at a highly probable location are reduced (Ferrante et al. 2018; Sha and Jiang 2016; Wang and Theeuwes 2018a, 2018b).

Similarly, other studies have demonstrated that experience with reward can have a persistent effect on selection. For instance, Anderson et al. (2011a) conducted an experiment consisting of a training and a test session. During training, participants searched for a red or a green circle presented among differently colored nontarget circles. One of the target colors was associated with high reward, while the other color was associated with low reward. During the subsequent test session, participants searched for a shape singleton presented among randomly colored nontarget shapes (additional singleton task; Theeuwes 1992). During this test session, one of the circles (which were now irrelevant for the task) had a color that was associated with high or low reward. Crucially, search time for the target increased when a nontarget had a color that was previously associated with a high reward, relative to when it was associated with a low reward. These findings suggest that reward-driven effects can persist over extended periods of time, and even take precedence over voluntary attentional control, even in conditions in which observers are explicitly indicated to ignore the reward-associated signals, or when they are well aware that these signals no longer predict any reward (Le Pelley et al. 2015; Pearson et al. 2015; Preciado et al. 2017).

Importantly, reward-associated stimuli have been shown to attract and capture attention in a rapid, automatic way, such that reward-driven attentional effects are visible even at very early stages of perceptual processing. For example, spatial cueing experiments using reward-associated stimuli have shown attentional costs and benefits, indicating that stimuli previously associated with reward summon attention to their location (Failing and Theeuwes 2014; Lynn and Shin 2015; Preciado et al. 2017). Similarly, stimuli examining the effect of reward on oculomotor performance have found similar effects in terms of eye movements, such that stimuli previously associated with reward not only captured attention but also attracted eye movements, even despite the fact that the stimulus reward contingency was no longer relevant (Hickey and van Zoest 2012, 2013; McCoy and Theeuwes 2016; Theeuwes and Belopolsky 2012). From this perspective, it can be concluded that reward may change the salience of a stimulus, such that it becomes more pertinent and, therefore, captures attention and eye movements in an automatic way, regardless of the strategic, voluntary goals of the observer.

The current study was designed to examine the extent to which reward-associated stimuli could elicit attentional and oculomotor effects comparable to those typically observed in the classic antisaccade task in the absence of any exogenous, stimulus-driven factors. Typically, the classic antisaccade task capitalizes on the fact that stimuli with an abrupt asymmetrical onset automatically and reflexively capture attention and eye movements, thus pitting this natural automatic bias against a voluntary, endogenous set, prompting the observer to look away from the abruptly appearing stimulus. This design has been instrumental to elucidate the interactions between exogenous, stimulus-driven biases on one hand, and goal-driven attentional and oculomotor control on the other (Hallett 1978; Hutton 2008; Munoz and Everling 2004). However, the particular features that make the classic antisaccade task an ideal paradigm to investigate the interaction between exogenous and endogenous biases makes it less adequate to investigate the interaction and competition between biases driven by selection history and endogenously controlled attention and eye movements, primarily due to the presence of abrupt asymmetric onsets. Indeed, if the classic antisaccade task were to be enhanced with the same reward manipulation that we have introduced in the present study, such an experiment would imply the interplay of the three described bias categories. Specifically, voluntary, endogenous attention (driven by the antisaccade instructions) would be competing with both automatic, exogenous attention (driven by the abrupt asymmetrical onsets), and selection history (driven by reward). Moreover, in such a design, exogenous and reward-driven factors would be congruent, pulling attention and eye movements in the same direction, making it impossible to disentangle the true effects of the each separate bias type.

Given that our objective is to investigate the interaction between selection history and endogenous attention in the absence of any exogenous stimulus-driven factors, it was necessary to effectively remove the influence of exogenous factors in our study, and thus, we introduced a few key modifications to the classic antisaccade task. Specifically, rather than using abrupt unilateral exogenous cues to examine the deployment of attention, we used bilateral cues (one colored and one gray) that were equally salient and always appeared simultaneously and at mirror locations on the display. A comparable strategy was employed by Theeuwes and Van der Stigchel (2006), in a study in which they demonstrated that faces, compared with non-faces, are more likely to summon attention and facilitate visual selection. Importantly, in a control study, pitting non-faces against inverted faces, they found no differences in saccade latency to either stimulus type, suggesting that the presentation of bilateral stimuli effectively abolished the influence of abrupt asymmetrical onsets on visual selection. Similarly, other studies have employed the same strategy to investigate the effect of stimuli associated with rewards or threats on spatial attention by means of a modified Posner cueing paradigm in which stimuli were presented bilaterally, and these have successfully revealed performance differences in perceptual sensitivity and response times that can be assumed to be driven purely by selection history (Preciado et al. 2016, 2017).

In addition, for the present study, the specific color of the cue was associated with different levels of reward during a training session. With this design, participants were presented with a task in which two equally salient stimuli were presented simultaneously on each trial. Participants were first trained to make a rapid and accurate saccade toward a colored stimulus that, depending on its color, was associated with a high reward, low reward, or no reward. Following training, during a testing phase, participants were instructed to make a saccade away from the colored, reward-associated stimulus, in line with the antisaccade task. With this experiment, we wanted to determine whether the typical findings reported for the classic antisaccade design (specifically, longer saccades to the target and an increased proportion of incorrect eye movements) could also be discovered in the absence of any strong exogenous pull by an abrupt onset stimulus. Consequently, we expect our modified paradigm to allow us to examine the extent to which the attentional effects elicited by selection history and reward against are comparable to those elicited by purely exogenous, stimulus-driven factors (see also Theeuwes, 2018 for similar arguments).

Notably, with this design, we can disentangle and separately examine the effects of factors crucial for the development of attentional and oculomotor biases driven by selection history: practice (understood as the repetition of the same oculomotor response over several trials within each phase of the experiment) and reward (Hikosaka and Isoda 2010; Isoda and Hikosaka 2007; Yamamoto et al. 2013). Specifically, this design allowed us to examine the effects of practice per se during the second phase of the experiment, in which no rewards are delivered—and, thus, any changes in performance can only result from repetition—and compare them to the effects of practice reinforced with reward during the first phase, by assessing the attentional effects elicited by cues associated with a high reward, low reward, or no reward. In addition, we investigated whether the effects observed were affected by individual differences. It is known that the ability to suppress automatic, prepotent responses is associated with differences in trait impulsivity (Aron and Poldrack 2005; Chambers et al. 2009; Congdon et al. 2012; Fillmore 2003; Logan et al. 1984). Individuals with high levels of impulsivity require more conscious effort to suppress prepotent responses, resulting in increased proportion of incorrect saccades and increased response times required to provide correct responses during an antisaccade task. To examine the extent to which individual differences influenced oculomotor performance in our task, we included the Barratt Impulsivity Scale-11 (BIS-11) (Patton et al. 1995; Stanford et al. 2009), a widely used self-reported questionnaire used to assess trait impulsivity.

## METHODS

### Participants

For this study, we tested 31 participants (24 female, 7 male) recruited from the Vrije Universiteit Amsterdam (mean age: 23.96 ± 3.16 yr old). All participants reported having normal or corrected-to-normal visual acuity, without colorblindness or any impairment in color vision. They also reported having no psychiatric, psychological, or neurological conditions, and signed a written informed consent before taking part in the study. For their participation, they received a monetary compensation of €9, plus a bonus contingent on their performance in the experimental task (maximum €5). All methods and procedures were approved by the Scientific and Ethical Review Committee of the Faculty of Behavioral and Movement Sciences of the Vrije Universiteit Amsterdam and are in line with the Declaration of Helsinki.

### Materials and Stimuli

The tasks used in this study were designed and conducted using OpenSesame version 3.1 (Mathôt et al. 2012) and the PyGaze toolbox (Dalmaijer et al. 2014). Participants were tested in a sound-attenuated, dimly lit cubicle; seated 75 cm away from a 22-inch computer screen (Samsung Syncmaster 2233RZ, 1,680 × 1,050 resolution, 120-Hz refresh rate) with their head positioned on a chin rest. Eye movements from the right eye were recorded through an EyeLink 1000 Tower-Mount eye-tracker system (SR Research, Oakville, ON, Canada), with 1,000-Hz temporal and <0.01° spatial resolution. Saccades were detected by an automatic algorithm using minimum velocity (35°/s) and acceleration (9,500°/s^2^) as detection criteria.

Stimuli consisted of isoluminant light-gray and colored circles (1.4° diameter) presented on a dark-gray background. These circles appeared simultaneously on every trial at each side of the central fixation cross (5.5° eccentricity). Colored circles could be blue, green, or orange, and they were all matched in luminance with the light gray (38 cd/m^2^). Counter-balanced across participants, each one of these three colors indicated that a high, low, or no reward could be obtained for a correctly directed saccade, and that the bonus payment was determined by the accumulated score. Note that the task was designed so that the colored circle was equally likely to appear at either side of the display and was always paired with a light-gray one. Trials including each color were intermixed, and every color was equally likely to appear on any given trial. Participants were instructed to make a saccade as quickly and accurately as possible toward one of these circles (target) while ignoring the other (distractor). The specific target stimulus depended on the phase of the experiment: during *phase I*, the target was the colored circle, and during *phase II*, it was the light-gray one (see *Procedure* below for details). In addition to the eye-tracking task, to explore the relationship between reward, oculomotor control, and individual differences in impulsivity as a personality trait, participants completed the BIS-11 (Patton et al. 1995; Stanford et al. 2009).

### Procedure

After signing the informed consent, the session started with the administration of the BIS-11 questionnaire. Following this, participants were instructed on the details of the task verbally and in written form. Specifically, they were informed that the task conditions and instructions were different for each phase in terms of the identity of the target and distractor stimuli and the availability of reward: in *phase I*, participants were told to make a fast, direct eye movement toward the colored stimulus. During this phase, each correct saccade granted points, depending on the color presented on the screen: 10 points for a high-reward color, 1 point for low-reward, and 0 for no reward. The score during the first phase determined their reward, up to a maximum of €5. Note that the participants were informed that different colors were associated with different levels of reward, but the specific color-reward associations had to be discovered through direct experience during the task. Conversely, during *phase II*, participants were instructed to make an eye movement toward the gray stimulus. Moreover, it was explicitly indicated that they could no longer earn points during this phase, and thus, their performance on the second part of the task would not affect their payout. Just before starting the task, each participant completed a 9-point calibration and validation procedure, and each phase began with automatic drift correction.

**Fig. 1.**
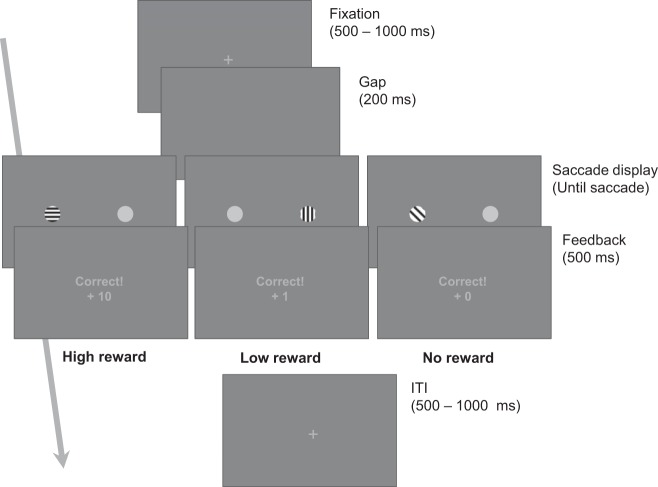
Task display sequence and trial types. In this image, colors are represented as lined circles for the sake of clarity, but solid colors were used on the actual experiment. Note that reward-color associations were counter-balanced across participants, colored circles were equally likely to appear at either side of the saccade display, and trial types were intermixed within blocks, all equally likely to occur on any given trial.

In both phases, to start a trial, participants were instructed to look at a fixation cross at the center of the screen. A participant’s gaze had to be detected within an area of 2.7° around the central cross for 100 ms for the trial sequence to continue. After verifying that the gaze was fixated within this region, the fixation cross was kept in display for a variable interval between 500 and 1,000 ms and then removed. After this, an empty display that was presented for 200 ms, followed by the saccade display presenting colored and gray circles to the left and the right of the original fixation (Fig. 1). We included this delay between fixation and saccade displays to promote shorter saccade latencies, in line with studies investigating this phenomenon, known as the gap effect. These studies have established that saccade latencies are shorter when an empty display is presented for a brief duration (~200 ms) between the offset of a fixation and the onset of a target display (Bekkering et al. 1996; Kingstone and Klein 1993; Meeter et al. 2010; Munoz and Everling 2004; Saslow 1967).

Upon the presentation of the saccade display, participants made a fast, direct saccade toward the target stimulus (i.e., the colored or light-gray stimulus, depending on the phase of the experiment). Following the saccade, a feedback display was presented for 500 ms, indicating the accuracy on the present trial (correct/incorrect) and obtained points (during the prosaccade phase only). Correct saccades were defined in terms of saccade latency (initiated within 500 ms from the onset of the saccade display) and landing position (within a 2.7° area around the target stimulus), such that saccades longer than the maximum latency, or landing outside of the defined target region were reported to the participant as incorrect on the subsequent feedback display. The next trial started after an intertrial interval ranging between 500 and 1,000 ms, randomly chosen from a uniform distribution. Each phase of the experiment was divided in three blocks of 100 trials, and participants were allowed to take a break after every block, indicating via keypress whenever they were ready to move on to the next block.

### Data Analysis

Raw eye-tracking data was extracted using EyeLink’s Data Viewer software (version 2.6.58; SR Research). Data processing and analysis were conducted in R version 3.4.2 (R Core Team 2017). For all analyses, gaze coordinates were rereferenced, such that positive coordinates represent the target’s hemifield (regardless of phase), and negative ones represent the distractor’s. We considered only the first saccade made after the onset of the saccade display, excluding trials containing blinks and those in which the saccade landed away from the defined target or distractor regions. For all analyses, statistical significance was evaluated for α = 0.05.

#### First saccade.

The analysis of the full first saccades focused on the mean latency (defined as the lapse between the onset of the saccade display and the initiation of the first detectable saccade), and the proportion of saccades directed toward the distractor (error saccades) calculated for each participant, phase, and reward level. Mean latency was calculated including only correctly directed saccades. To inspect the development of a reward-driven bias over the course of the experiment, we also computed and evaluated mean latency and the proportion of error saccades for each one of the three blocks making up each phase to be able to use block as a factor in the statistical analysis to track the evolution of attentional effects as a function of reward and practice (i.e., repetition) within each phase of the experiment.

Additionally, we employed the Vincentizing procedure (Ratcliff 1979) to better understand the relationship between the latency and landing position (on target or distractor) of the first saccade, and the different reward levels and the phase of the experiment. To do this, we first determined the cumulative distribution function of saccade latencies for every participant and experimental condition to obtain the latency deciles of each distribution, and then computed the proportion of error saccades for the trials with saccade latencies corresponding to each of these deciles.

#### Statistical analysis.

Data analysis was conducted with 2 × 3 repeated-measures ANOVA approach, with phase (I and II) and reward (high, low, and no reward) as predictors. Additionally, in subsequent analyses we included block (three levels) as a factor to examine the evolution of attentional effects over the course of the experiment. Similarly, for the analysis of Vincentized data, we included latency decile as an additional factor to evaluate the relationship between saccade latency on landing position. For all analyses, the sphericity assumption was evaluated with Mauchly’s test, and whenever this assumption is violated, we applied the Greenhouse-Geisser *P* value correction, reporting uncorrected degrees of freedom for the sake of clarity. Further exploration of effects was conducted via paired, two-sided *t*-tests, including the false-discovery rate (FDR) correction for multiple comparisons. For each one of the *t*-tests, Cohen’s *d* was calculated as an estimate of effect size, computed as the *t-*statistic divided by the squared-root of the sample size (Lakens 2013).

### Impulsivity

To explore the relationship between individual differences in trait impulsivity and performance on the eye-tracking task, we calculated the Spearman ρ correlations between scores obtained from the self-report questionnaires on one side, and the collected first saccade measures (mean latency and proportion of error saccades at each phase and reward level).

## RESULTS

Data from one participant with performance below 75% correct on the first phase of the experiment was excluded from the analysis; thus, the presented results are based on the remaining 30 participants (24 female, 6 male; mean age: 23.83 ± 3.13 yr old). Trials with missing coordinates due to blinks or eye-tracking artifacts (32 trials, 0.18% data loss), and trials in which the latency of the first saccade was shorter than 80 ms (349 trials, 1.94% data loss) or longer than 500 ms (154 trials, 0.86% data loss) were excluded from further analyses. We also discarded trials with gaze samples falling outside the screen range (33 trials, 0.18% data loss), and trials in which the origin or landing positions of the first saccade were outside an area of 2.7° around the central fixation or the stimuli locations, respectively (675 trials, 3.75% data loss). All analyses are based on the remaining 16,757 trials. Mean and standard deviations for saccade latency to targets and distractors, as well as the proportion of error saccades, are presented in Table 1.

**Table 1.** Descriptive statistics of saccade latency and proportion of error saccades for each phase (I and II) and reward level (high, low, and no reward)

	High Reward		Low Reward		No Reward	
	*Phase I*	*Phase II*	*Phase I*	*Phase II*	*Phase I*	*Phase II*
*n* (Trials)	2.817	2.764	2.821	2.769	2.795	2.791
Saccade latency to target, ms	202.78 (51.28)	205.60 (51.62)	211.58 (56.85)	204.09 (51.29)	210.34 (54.21)	204.95 (52.13)
Saccade latency to distractor, ms	164.98 (42.57)	163.20 (48.28)	178.17 (49.87)	152.79 (33.95)	174.96 (38.83)	155.76 (40.73)
Error saccades	2.63%	9.44%	3.05%	6.97%	3.51%	7.78%

Values are expressed as means (SD).

### First Saccade

#### Saccade latency to target.

The repeated-measures ANOVA conducted on saccade latency showed reliable main effects of reward [*F*(2, 58) = 5.708, *P* = 0.005, $\eta_{\text{p}}^{2}$ = 0.001], and the interaction between phase and reward [*F*(2, 58) = 16.015, *P* < 0.001, $\eta_{\text{p}}^{2}$ = 0.004], but not for phase [*F*(1, 29) = 1.038, *P* = 0.32]. Further examination of these effects revealed that saccade latencies were significantly shorter on high-reward trials, in contrast to both low [*t*(29) = 2.94, *P* = 0.02, Cohen’s *d* = 0.54], and no reward [*t*(29) = 3.70, *P* = 0.003, Cohen’s *d* = 0.68]. No differences in saccade latency were observed between low and no reward [*t*(29) = 0.18, *P* = 0.86].

**Fig. 2.**
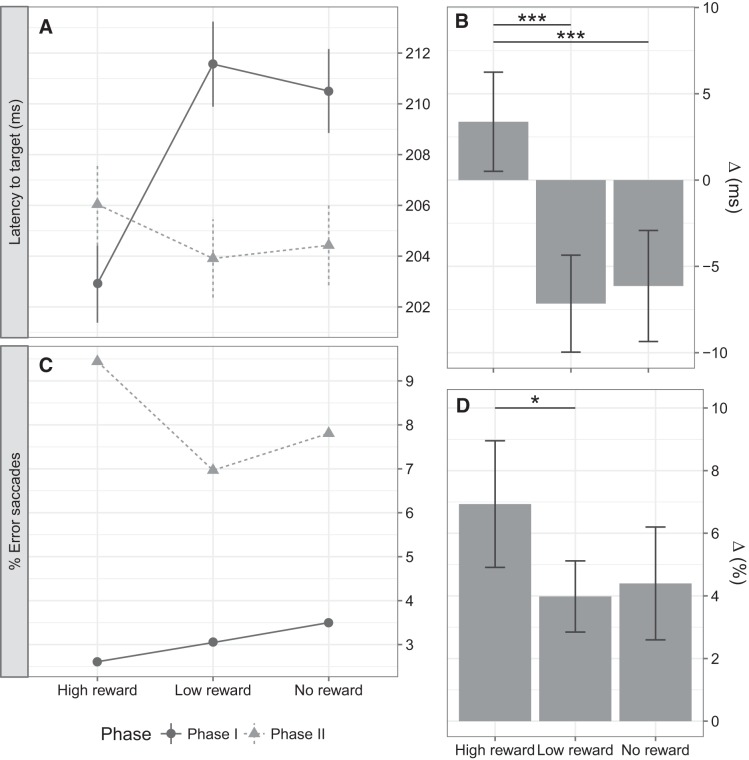
Phase × Reward interactions. *A* and *C*: latency of saccades made to the target (i.e., correct saccades only) and % of error saccades (respectively) by phase and reward level. *B* and *D*: computed differences in latency and % of error saccades (*phase II* and *phase I*). Error bars represent within-subjects confidence intervals (Morey 2008). Lines emphasizing significant effects represent *t*-tests results on the computed differences. ***P ≤ 0.001; *P ≤ 0.05.

The analysis of the interaction between phase and reward indicates that the latency differences between reward levels was present only during *phase I*, producing significantly shorter saccades on high-reward trials compared with low [*t*(29) = 4.73, *P* < 0.001, Cohen’s *d* = 0.86] and no reward [*t*(29) = 4.68, *P* < 0.001, Cohen’s *d* = 0.85] (Fig. 2*A*), with no differences between reward levels on *phase II*. To further investigate this interaction, we computed the difference in saccade latencies between phases for each reward level and compared these difference scores, revealing a clear contrast between latency changes between phases in high-reward trials, as compared with low- [*t*(29) = 5.64, *P* < 0.001, Cohen’s *d* = 1.02] and no-reward trials [*t*(29) = 4.40, *P* < 0.001, Cohen’s *d* = 0.80]. These differences indicate that saccades to high-reward targets became significantly longer during the second phase of the experiments, while saccade latencies on both low- and no-reward targets actually became shorter (Fig. 2*B*).

**Fig. 3.**
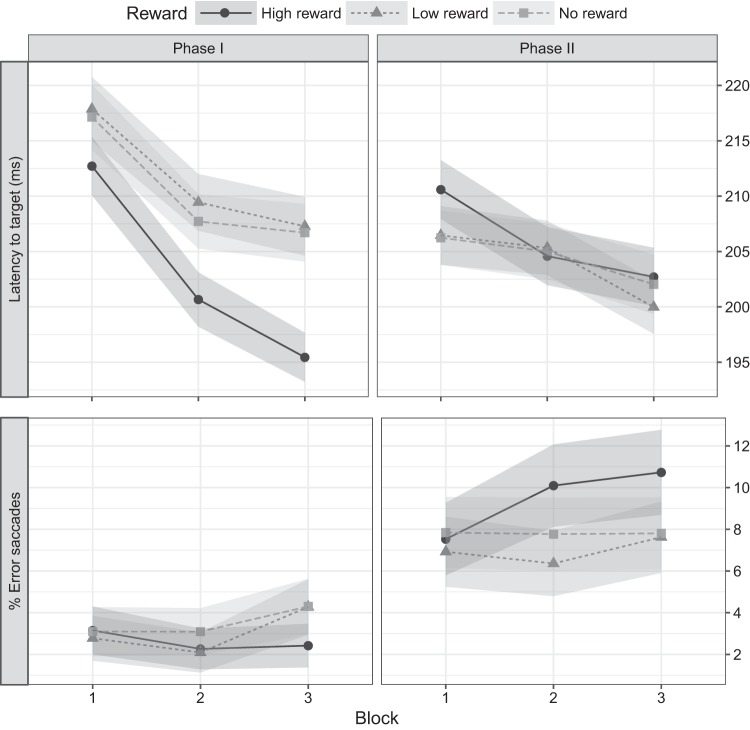
Phase × reward × block interactions. *A* and *B*: presents the latency of saccades made to the target (i.e., correct saccades only) and % of error saccades (respectively) by phase, reward level, and block. Shaded areas represent within-subject confidence intervals (Morey 2008).

Including block as a factor in the analysis replicated the main effect of reward [*F*(1, 29) = 5.4187, *P* = 0.007, $\eta_{\text{p}}^{2}$ = 0.13] and the interaction between phase and reward [*F*(1, 29) = 14.7946, *P* < 0.001, $\eta_{\text{p}}^{2}$ = 0.036] observed in the previous analysis. Moreover, it revealed a main effect of block [*F*(1, 29) = 24.6515, *P* < 0.001, $\eta_{\text{p}}^{2}$ = 0.09], consistent with an expected gradual reduction of saccade latencies as the experiment progressed and the participants became more proficient in the task (Fig. 3*A*). In addition, the two-way interaction between block and reward was also reliable [*F*(2, 58) = 4.0461, *P* = 0.023, $\eta_{\text{p}}^{2}$ = 0.0032]. Comparisons between reward levels within each block (averaged over phase) showed no latency differences between reward levels during the first block (*P* > 0.05 for all paired *t-*tests), yet latencies for high-reward trials were significantly shorter than both low- and no-reward trials for the second (high vs. low *t*(29) = 3.14, *P* = 0.009, Cohen’s *d* = 0.57; high vs. no *t*(29) = 2.61, *P* = 0.028, Cohen’s *d* = 0.48) and third blocks (high vs. low *t*(29) = 3.14, *P* = 0.009, Cohen’s *d* = 0.57; high vs. no *t*(29) = 4.36, *P* < 0.001, Cohen’s *d*  0.78), indicating the gradual reduction of saccade latencies was more pronounced for high-reward signals. No differences were found between low- and no-reward trials within any block (FDR-corrected *P* > 0.05 for all paired *t-*tests).

#### Proportion of error saccades.

The 2 × 3 repeated-measures ANOVA calculated on the proportion of error saccades revealed a significant main effect of phase [*F*(1, 29) = 31.871, *P* < 0.001, $\eta_{\text{p}}^{2}$ = 0.21]; and a significant interaction of phase and reward [*F*(2, 58) = 3.711, Greenhouse-Geisser corrected *P* = 0.04, $\eta_{\text{p}}^{2}$ = 0.02], but no main effect of reward [*F*(2, 58) = 2.072, *P* = 0.14]. The effect of phase reflected the expected increase in the proportion of saccades toward the distractor during *phase II* [*t*(29) = 5.59, *P* < 0.001, Cohen’s *d =* 1.02]. We first examined the interaction between phase and reward by comparing the proportion of error saccades between each level of reward within each phase of the experiment. These contrasts did not reveal any statistically reliable differences between reward levels within either phase. Nevertheless, we found a marginally significant difference between high- and low-reward trials during *phase II* [*t*(29) = 2.36, *P* = 0.67], consistent with the notion that stimuli associated with high reward are, indeed, more likely to attract and capture attention, leading to incorrect eye movements. Together, the presented results indicate that the increase in error saccades on *phase II* was consistent on every level of reward (high: *t*(29) = 6.32, *P* < 0.001, Cohen’s *d =* 1.15; low *t*(29) = 3.67, *P* = 0.004, Cohen’s *d =* 0.67; no: *t*(29) = 3.65, *P* = 0.004, Cohen’s *d =* 0.67), in concordance with the reported main effect of phase (Fig. 2*C*).

To further examine this interaction, we again calculated the difference in proportion of error saccades between phases and compared these difference scores between reward levels. This comparison revealed a significant difference between high and low reward (*t*(29) = 2.70, *P* = 0.037, Cohen’s *d =* 0.49), indicating that the increase in error saccades on the second phase of the experiment was greater for high-reward trials than for low-reward ones. No statistical differences were observed between low and no reward [*t*(29) = 0.47, *P* = 0.64], or between high and no reward [*t*(29) = 1.74, *P* = 0.20] (Fig. 2*D*).

Including block in the analysis of the proportion of error saccades replicated the significant main effect of phase [*F*(1, 29) = 31.1210, *P* < 0.001, $\eta_{\text{p}}^{2}$ = 0.26] and the interaction between phase and reward [*F*(2, 58) = 3.8928, *P* = 0.026, $\eta_{\text{p}}^{2}$ = 0.024]. In addition, it revealed a significant three-way interaction between phase, reward, and block [*F*(2, 58) = 3.3867, *P* = 0.041, $\eta_{\text{p}}^{2}$ = 0.011, Fig. 2*C*]. None of the other main effects or interactions was statistically reliable. Similarly, paired *t*-tests conducted to further explore the significant three-way interaction did not reveal any differences in the proportion of error saccades between any of the evaluated conditions (all *P* > 0.05).

#### Vincentized proportion of error saccades.

The ANOVA analysis adding latency decile as a factor with the proportion of saccades to the distractor at each of the calculated deciles as dependent variable replicated the significant main effects of phase [*F*(1, 29) = 31.622, *P* < 0.001, $\eta_{\text{p}}^{2}$ = 0.14] observed for the proportion of error saccades, and revealed a main effect of saccade latency decile [*F*(1, 29) = 50.699, *P* < 0.001, $\eta_{\text{p}}^{2}$ = 0.37], suggesting that the direction of a saccade is strongly influenced by how fast the saccade is initiated but did not reveal any effect of reward [*F*(2, 58) = 2.1, *P* = 0.13]. The two-way interactions between phase and reward [*F*(2, 58) = 3.692, *P* = 0.031, $\eta_{\text{p}}^{2}$ = 0.011] and between phase and latency decile [*F*(1, 29) = 36.992, *P* < 0.001, $\eta_{\text{p}}^{2}$ = 0.18] were also found to be statistically significant. In contrast, the interaction between reward and latency decile [*F*(2, 58) = 2.037, *P* = 0.14] or the three-way interaction between phase, reward, and latency decile [*F*(2, 58) = 2.942, *P* = 0.061] did not reveal any reliable effects.

**Fig. 4.**
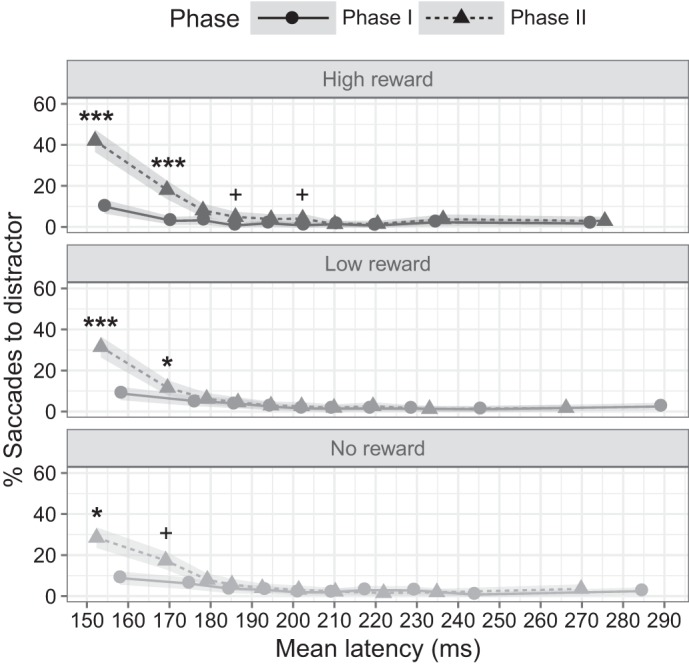
Vincentized proportion of saccades to distractor for each phase (solid and dashed lines for *phase I* and *II*, respectively) and reward level. Shaded regions correspond to within-subject confidence intervals (Morey 2008). Each data point represents a decile in the corresponding latency cumulative distribution. ***P ≤ 0.001; *P ≤ 0.05; +*P* ≤ 0.06.

These results indicate that the landing position of a saccade is strongly influenced by the how fast the saccade is initiated, in addition to the phase and reward level of the experiment. Paired *t-*tests contrasting the proportion of error saccades for every decile and reward level between phases of the experiment indicated that the increase in saccades toward the distractor on *phase II* was driven primarily by the saccades with the shortest latency (Fig. 4). Specifically, these comparisons indicate that, on high-reward trials, the increase in the proportion of error saccades was statistically reliable in the 1st [*t*(29) = 6.38, *P* < 0.001, Cohen’s *d =* 1.16] and 2nd deciles [*t*(29) = 4.90, *P* < 0.001, Cohen’s *d =* 0.89], and marginally significant on the 4th [*t*(29) = 2.67, *P* = 0.053, Cohen’s *d =* 0.49] and 6th deciles [*t*(29) = 2.59, *P* = 0.056, Cohen’s *d =* 0.47], suggesting that, during *phase II*, a substantial proportion of the saccades with the shortest latency on high-reward trials was more likely to be directed toward the distractor than the target, in contrast to the other reward conditions. Indeed, low-reward trials only showed this bias on the 1st decile [*t*(29) = 4.66, *P* < 0.001, Cohen’s *d =* 0.85] and 2nd decile [*t*(29) = 2.84, *P* = 0.048, Cohen’s *d =* 0.51]; and no-reward trials showed it exclusively on the 1st [*t*(29) = 5.1, *P* < 0.001, Cohen’s *d =* 0.93], and marginally for the 2nd decile [*t*(29) = 2.71, *P* = 0.053, Cohen’s *d =* 0.49].

### Impulsivity

Trait impulsivity, as evaluated by the BIS-11 instrument (Patton et al., 1995; Stanford et al., 2009), is not understood as a single, unified concept, but rather as a construct that integrates cognitive and behavioral components. Specifically, the BIS-11 operationalized impulsivity in terms of three dimensions (second-order factors), each one, in turn, composed of second subdimensions (first-order factors). High scores in either of these are considered a manifestation of higher levels of trait impulsivity in an individual. For our study, we will focus only on the second-order factors, namely motor impulsivity, understood as acting without forethought; cognitive/attentional impulsivity, involving rapid, reckless decision making and inability to concentrate; and nonplanning, understood as a lack of forethought.

### Impulsivity and Saccade Latency

The analysis of the Spearmanʼs ρ correlations between mean saccade latency (for each phase and reward level) and impulsivity scores (second-order dimensions) revealed only a significant negative correlation between motor impulsivity and saccade latency on trials with no reward during *phase II* (ρ = −0.41, *P* = 0.023), indicating that people with higher motor impulsivity scores made saccades toward targets not signaling reward with significantly shorter latencies. Note that all computed correlations reflect the same pattern, namely, higher motor impulsivity scores associated with shorter saccade latencies for all levels of reward, yet for the present data, only the correlations involving no reward trials reached statistical significance.

### Impulsivity and Proportion of Error Saccades

**Fig. 5.**
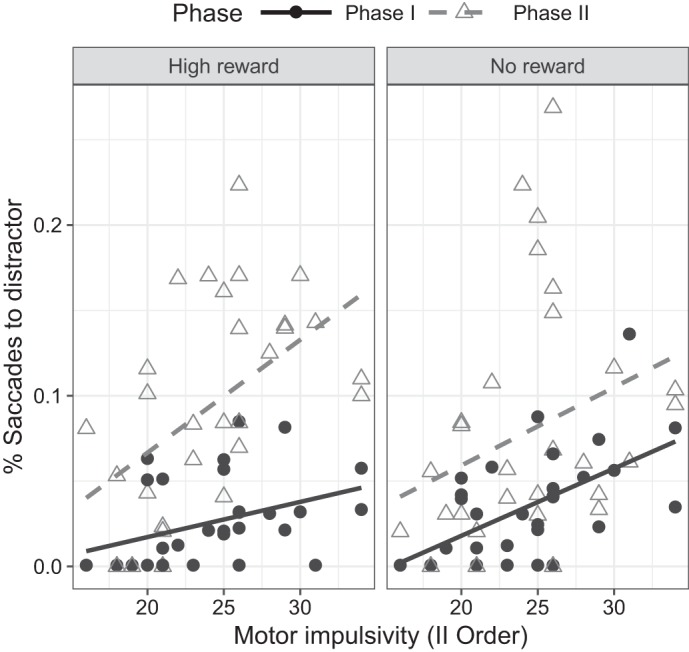
Spearman ρ correlations between second-order motor impulsivity and the proportion of saccades to the distractor by phase and reward (low reward data not shown, as it did not significantly correlate with motor impulsivity).

In terms of the proportion of error saccades, the analysis of correlations revealed a number of significant associations clustered on the motor impulsivity dimension. Specifically, this analysis revealed reliable associations between motor impulsivity and the proportion of incorrect responses on high-reward trials during both phases of the experiment (*Phase I*: ρ = −0.44, *P* = 0.016; *Phase II*: ρ = −0.58, *P* < 0.001), as well as on no reward trials (*Phase I*: ρ = −0.55, *P* = 0.002; *Phase II*: ρ = −0.42, *P* = 0.019). These results indicate that higher scores on motor impulsivity are associated with an increased proportion of saccades falling on the distractor, rather than on the target. Moreover, the observed pattern of responses indicates that, for individuals with high scores in motor impulsivity, the performance decrease observed between *phase I* and *II* is more dramatic for high-reward trials, in contrast to no reward, suggesting that automatic oculomotor response to high-reward trials developed over *phase I* might be more difficult to overcome for individuals with higher scores on motor impulsivity (Fig. 5).

## DISCUSSION

In this study, we employed a modified version of the classic antisaccade task to investigate the effect of stimuli associated with different levels of reward on oculomotor control. Specifically, we wanted to establish whether specific reward associations acquired over the course of several trials would elicit oculomotor and attentional effects comparable to those typically reported for the antisaccade task, namely, longer saccade latencies and increased proportion of saccades to the distractor stimuli on *phase II* trials, compared with *phase I*. In contrast to previous studies using the antisaccade task in which there are always stimuli with an abrupt onset, our experiment controlled for the influence of exogenous, stimulus-driven effects, allowing us to establish whether selection history, in the form of learned stimulus-reward associations, elicited similar oculomotor and attentional effects than those typically attributed to automatic, stimulus-driven attention.

Our analysis of the first saccades revealed a reliable effect of phase on the proportion of error saccades, but not for saccade latencies toward the target. The effect of phase provides a measure of the control conflict between voluntary and automatic, reward-driven oculomotor biases, and as such, our findings are congruent with the well-documented increase in the proportion of errors typically reported during in the antisaccade task (Hallett, 1978; Hutton, 2008). Crucially, the analyses revealed reliable interactions between phase and reward for both saccade latencies and the proportion of error saccades, indicating that the expected differences in oculomotor performance between the phases of the experiment was modulated by the level of reward.

Specifically, during *phase I*, we observed that saccade latencies to high-reward trials were noticeably shorter than those on both low- and no-reward trials. Similarly, the proportion of error saccades during this phase was the lowest for high-reward trials. Together, these results indicate that the learned stimulus-reward associations led to a response bias that optimized responses to high-reward trials. These results suggest that all participants were, indeed, able to learn the specific reward contingencies and that such learning selectively biased attention and oculomotor performance on high-reward trials during the prosaccade phase. These findings are consistent with existing reward literature, confirming that stimuli associated with reward lead to the development of a selection bias, which results in optimized performance in detecting and responding to these stimuli (Anderson et al. 2011a; Folk et al. 1992; Stankevich and Geng 2014).

Combined, these results from *phase I* highlight the role of two key factors underscoring the development of attentional and (oculo)motor biases driven by selection history: Practice (i.e., repetition) and reinforcement, in the form of reward in this case (Bentley et al. 2003; Della Libera and Chelazzi 2006; Yamamoto et al. 2013). Indeed, the differences we observed in latency and proportion of correct saccades between high reward on one hand and low and no reward conditions on the other can be taken to reflect the additivity of the effects of reinforcement and repetition (Engelmann et al. 2009; Stankevich and Geng 2014). In our design, stimuli associated with every level of reward were presented for an equal number of trials within every 300-trial block, implying that the amount of practice with each of the stimuli was the same. Yet, our results revealed noticeable performance differences favoring high reward over the other reward levels, further validating the notion that reward and repetition additively contribute to the development of the attentional bias (Hikosaka and Isoda 2010; Isoda and Hikosaka 2007; Stankevich and Geng 2014; Yamamoto et al. 2013). This interpretation is further strengthened by the inclusion of block as a factor in our analyses, which revealed that saccade latencies to high-reward trials became notably shorter, but only starting from the second block of *phase I*.

Interestingly, while the described results are largely consistent with those obtained with the classic antisaccade task, there are certain differences that should be considered. First, we did not observe a statistically significant difference in saccade latency between experimental phases, even though such a difference is often reported in the classic antisaccade literature. Similarly, the effects that we observed in our experiment are comparatively smaller than those reported by these studies (Hallett 1978; Hutton 2008). Lastly, we found that saccade latencies on high-reward trials were significantly slower during the second phase of the experiment, compared with the first. In contrast, saccade latencies to low- and no-reward trials became consistently faster.

The fact that we did not replicate a highly reliable phase effect in our task and that the size of our effects is comparatively smaller than those reported with the standard antisaccade studies may result from the crucial differences between our task and the classic antisaccade. Critically, the classic antisaccade task employs a highly salient, asymmetrical stimulus to automatically and exogenously summon attention. In our experiment, we wanted to eliminate the effect of exogenous capture by means of symmetrical stimuli with an abrupt onset, to examine the effect of selection history. Stimulus-driven (exogenous) factors have been demonstrated to be powerful modulators of attentional and oculomotor selection that are very difficult to suppress (Godijn and Theeuwes 2002; Theeuwes 1991), and thus, it is plausible that the comparatively smaller effect size and the absent significant main effect of phase on saccade latency reflect the difference between exogenous and selection history-driven attention.

Regarding the paradoxical shortening of saccade latencies on low- and no-reward trials during *phase II*, while inconsistent with well-known standard antisaccade effects, such a reduction in latencies is not an entirely novel finding. Indeed, it has been observed before in a study investigating the effect of task switching on antisaccade performance (Cherkasova et al. 2002). In this study, the authors compared antisaccade latency between a mixed condition, in which prosaccade and antisaccade trials are intermixed within a block; and a blocked condition, in which prosaccade and antisaccade trials were arranged in separate blocks. With this design, they observed a reduction in antisaccade latencies in the blocked condition, but not in the mixed blocks. The authors propose two alternative explanations for this discrepancy: First, it is argued that it results from intertrial effects, such that detrimental antisaccade effects propagate from one trial to the next in the mixed condition. Alternatively, the observed discrepancy can also result from endogenous facilitation, such that voluntary cognitive control makes response execution easier in the blocked condition. The authors further point out that further research would be necessary to clarify the origins of the observed reduction in saccade latencies.

Within the context of the present study, we interpret such paradoxical results to reflect the additive effect of reward and practice in the development of an oculomotor bias. In concrete, this pattern of results would suggest that, in the absence of reward and stimulus-driven biases, oculomotor responses can be adapted rapidly and flexibly to meet the demands of the task, since there is no other conflicting bias interacting with voluntary control and, thus, affecting performance. In other words, performance in these trials would depend solely on voluntary oculomotor guidance and, consequently, would not show any of the standard antisaccade effects, as indicated by earlier studies employing bilateral stimuli on a visual selection task (Theeuwes and Van der Stigchel 2006). Nevertheless, this is not the case for high-reward trials, in which the longer latencies typical of the antisaccade task are taken to reflect how the reward-association, combined with practice, has led to the development of an oculomotor bias that automatically summons attention to the reward-associated stimulus. This association, in turn, results in a delay in voluntary oculomotor responses, consistent with the expected conflict between automatic and voluntary oculomotor programs underlying the effects typically observed in the antisaccade task.

This notion is further supported by the results observed when block was included as a factor in our statistical analyses: while saccade latencies for low- and no-reward trials progressively and consistently became faster across phases as a function of practice, we observed a noticeable difference in saccade latencies for high-reward trials between the last block of the first phase and the first block of the second. Specifically, our results indicate that saccade latencies on high-reward trials were the fastest at the end of the first phase, but also the slowest at the end of the second phase, closely resembling the results from the classic antisaccade task. Together, we take the observed differences between the presented results and those from the classic antisaccade task to reflect the interplay between a learned selective, automatic bias in favor of the high-reward stimulus, and voluntary oculomotor guidance. In fact, this conflict is very similar to the conflict between automatic and voluntary control seen in classic antisaccade effects. Crucially, however, in the classic antisaccade task, automatic oculomotor control is driven by the physical properties of the stimulus in an exogenous fashion, while in our study, these automatic biases have been developed through reward and selection history.

The analysis of the results of the second phase not only replicated (in high-reward trials) the expected deficits in latency and accuracy typically reported in the antisaccade literature (Hallett 1978; Hutton 2008; Munoz and Everling 2004), but also indicates that reward-driven biases developed over the course of *phase I* persisted and interfered with performance on *phase II*, despite the fact that participants were well aware that no rewards were available during this phase. Indeed, our findings indicate that the increase in proportion of error saccades for high-reward trials was significantly higher than for low-reward trials, suggesting that the stimulus associated with high reward is more likely to attract and capture saccades, regardless of the intentions of the observer or whether reward is at all available (Anderson et al. 2011b; MacLean et al. 2016; Pearson et al. 2015). A comparable pattern was observed for saccade latency, such that correctly directed saccades on trials in which the stimulus associated with high reward became significantly longer, while saccades on trials presenting the other stimuli actually became shorter. These findings suggest that participants need to make an additional effort to suppress the prepotent automatic orienting response to high-reward trials. This, in turn, implies that the learned high-reward association made it more difficult for participants to suppress a saccade, and consequently, providing a correct response during these trials involved additional effort and processing steps, thus delaying the saccade.

Moreover, the analysis of the proportion of error saccades as a function, phase, reward, and saccade latency decile (Vincentization procedure) provided additional insights of interplay between selection history and voluntary oculomotor selection. Our findings indicate that, during *phase II*, saccades with short latencies are more likely to be directed toward the colored stimulus, reflecting the bias developed during the prosaccade phase. This result is similar to a study conducted by van Zoest et al. (2004), in which the extent to which saccadic visual selection depended on stimulus-driven or goal-driven biases was investigated, and they demonstrated that a critical factor in determining the dominant attentional bias was the latency of the saccade, such that the saccades with the shortest latency are largely determined by the stimulus-driven bias, while the longer ones reflected voluntary attentional control.

In this sense, our findings suggest that, much like with exogenous attentional biases, selection history biases determine the landing position of a saccade rapidly and automatically and that this tendency can only be overcome on longer saccades. Moreover, differences between reward levels on the Vincentized data once again revealed a selective bias for high-reward trials, which led to a significantly higher proportion of saccades with short latencies being directed to the distractor (up to more than 40% for high-reward trials at the decile corresponding to the shortest saccade latencies, compared with ~30% for the other levels of reward at the same decile). Similarly, we also found that, a greater proportion of the trials with the shortest saccade latency seems to be affected by the reward-driven oculomotor bias on high-reward trials, such that saccades with a latency falling within the first six deciles show a significant increase of error saccades, while this increase is only significant for the first two deciles for low- and no-reward trials. As a whole, these findings add up to confirm that selection history biases do elicit attentional and oculomotor effects paralleling those triggered by physically salient stimuli, suggesting that selection history effects can be elicited in a reflexive, automatic way.

Examining the extent to which trait impulsivity modulated the observed results revealed that participants reporting higher levels of motor impulsivity were more likely to make incorrect saccades during *phase II*, and showed also shorter saccade latencies. Combined, these results are consistent with the notion of individuals with high scores on impulsivity find it more difficult to suppress and overcome automatic, prepotent responses (Abroms et al. 2006; Aron and Poldrack 2005; Fillmore 2003). Interestingly, the neural underpinnings of this association between self-reported impulsivity and the observed deficiencies in suppressing prepotent responses have been examined using the same instrument that we used to assess impulsivity in our study (BIS-11; Patton et al. 1995) in a study by Buckholtz et al. (2010). In this study, the authors examined the hypothesis that individuals with higher scores in self-reported impulsivity show abnormalities in dopaminergic neurotransmission within striato-cortical circuits, specifically, on pathways originating from the ventral striatum (Substantia nigra and ventral tegmental area) targeting frontal cortical areas. This study demonstrated how highly impulsive individuals appear to suffer from disinhibited dopaminergic signaling from the ventral striatum to fronto-cortical terminal fields, leading to stronger neural responses toward rewarding, novel, or salient stimuli. On the basis of these findings, the authors conclude that abnormalities in the ascending dopaminergic pathways are associated with impulse control deficits. From this perspective, it can be inferred that the extent to which attentional and oculomotor control is determined endogenously or by value-driven biases is associated with trait impulsivity and that at individual differences in dopaminergic signaling lie at the heart of this association. Such an association can be very relevant for the investigation of impulse control and to understand the pathological mechanisms behind mental disorders for which trait impulsivity is a risk factor, such as addiction and behavioral control disorders. Similarly, it underscores the potential of the antisaccade task as a tool to predict the risk for developing psychiatric disorders (Hutton and Ettinger 2006; Mazhari et al. 2011).

In summary, our study provides evidence indicating that selection history biases can elicit attentional and oculomotor effects comparable to those triggered exogenously by physically salient stimuli, and that these effects are elicited in an automatic, reflexive manner. Furthermore, our findings are consistent with the notion that voluntary, goal-driven behavior can become automatized through practice and repetition, and that this process is facilitated by reward. We also show that reward-driven biases, once learned and consolidated, seem to require additional effort to be overridden, even if it is explicitly clear for the individual that the response-reward contingency is no longer valid. Moreover, we observed that the effects of these acquired reward-driven biases are sensitive to individual differences in trait impulsivity, such that more impulsive individuals will find it more difficult to overcome the effects of automatic reward-driven biases, indicating that their attention and eye movements are even more likely to be captured by reward-signaling stimuli, regardless of their voluntary intentions. These findings provide additional insights into the mechanisms underlying the development of selection history biases, indicating that automatic, reflexive effects are not exclusively triggered by physically salient stimuli, but can also be developed and maintained purely through selection history.

## GRANTS

This research was supported by an ERC Advanced Grant (ERC-2012-AdG-323413 to J. Theeuwes).

## DISCLOSURES

No conflicts of interest, financial or otherwise, are declared by the authors.

## AUTHOR CONTRIBUTIONS

D.P. and J.T. conceived and designed research; D.P. performed experiments; D.P. analyzed data; D.P. and J.T. interpreted results of experiments; D.P. prepared figures; D.P. drafted manuscript; D.P. and J.T. edited and revised manuscript; D.P. and J.T. approved final version of manuscript.
